# The origin of neutron biological effectiveness as a function of energy

**DOI:** 10.1038/srep34033

**Published:** 2016-09-22

**Authors:** G. Baiocco, S. Barbieri, G. Babini, J. Morini, D. Alloni, W. Friedland, P. Kundrát, E. Schmitt, M. Puchalska, L. Sihver, A. Ottolenghi

**Affiliations:** 1 Department of Physics, University of Pavia, Pavia, Italy; 2 INFN, National Institute of Nuclear Physics, Sezione di Pavia, Pavia, Italy; 3 LENA, Laboratory of Applied Nuclear Energy, University of Pavia, Pavia, Italy; 4 Institute of Radiation Protection, Helmholtz Zentrum München – German Research Center for Environmental Health, Neuherberg, Germany; 5 Technische Universität Wien, Wien, Austria

## Abstract

The understanding of the impact of radiation quality in early and late responses of biological targets to ionizing radiation exposure necessarily grounds on the results of mechanistic studies starting from physical interactions. This is particularly true when, already at the physical stage, the radiation field is mixed, as it is the case for neutron exposure. Neutron Relative Biological Effectiveness (RBE) is energy dependent, maximal for energies ~1 MeV, varying significantly among different experiments. The aim of this work is to shed light on neutron biological effectiveness as a function of field characteristics, with a comprehensive modeling approach: this brings together transport calculations of neutrons through matter (with the code PHITS) and the predictive power of the biophysical track structure code PARTRAC in terms of DNA damage evaluation. Two different energy dependent neutron RBE models are proposed: the first is phenomenological and based only on the characterization of linear energy transfer on a microscopic scale; the second is purely *ab-initio* and based on the induction of complex DNA damage. Results for the two models are compared and found in good qualitative agreement with current standards for radiation protection factors, which are agreed upon on the basis of RBE data.

The property of ionizing radiation in inducing early and late biological effects can be ultimately traced back to physical energy depositions at the cellular and sub-cellular scale. Such energy depositions proceed through electronic interactions giving rise to ionizations, thus disrupting the atomic or molecular structure of the absorber and producing chemical and biological damage. This is equally true when the primary particle traversing the biological structure is neutral: indirectly ionizing radiations (such as photons or neutrons) interact in such a way that their energy is transmitted accelerating directly ionizing secondary charged species^1^.

Neutrons in particular undergo a large variety of nuclear reactions in the biological target^2^, thus producing a mixed field of secondary charged particles. Nuclear reaction cross sections are strongly dependent on neutron energy, and so is the secondary particle field. Furthermore, since neutrons can be moderated in their interactions within the material, losing only part of their energy in a collision, the geometry of the receptor plays an important role: new reaction channels open when neutron energy changes, and different secondary particles can be produced at different depths in the target. To interpret the outcome of a radiobiological measurement of neutron effectiveness, it is therefore essential to take into consideration the characteristics of the exposure, namely: (i) neutron energy spectrum; (ii) how such spectrum is eventually modulated when neutrons traverse the biological target for given target dimensions.

In order to compare the effectiveness of different radiation qualities, the so-called Relative Biological Effectiveness (RBE) is introduced, traditionally defined as the ratio of the absorbed dose of a reference low linear energy transfer photon beam (as *e*.*g*. ^60^Co or 250 keV photons) to the absorbed dose of the test radiation type required to cause the same level of biological effect. RBE values obtained in radiobiological measurements depend on a large variety of factors, such as dose, dose-rates, investigated endpoint, and chosen experimental setups (*e*.*g*. cell lines for *in vitro* studies)^1^.

Pooling together RBE data from different experiments lay at the basis of the establishment of radiation protection standards, namely radiation weighting factors w_R_, which are then used to convert the physical absorbed dose (Gy) into an equivalent dose (Sv). Such weighting factors are given, for simplicity, irrespective of endpoint, tissue, dose rate, mode and heterogeneity of the exposure. Values for w_R_ are agreed upon by international regulatory commission as the International Commission on Radiological Protection - ICRP, based on maximum RBE values at low doses, which in turn depend on the assumptions about the low-dose shape of the dose-response curves for the test and reference radiations. Given the underlying simplifying assumptions, ICRP clearly states that the w_R_ formalism has to be adopted only for radiation protection purposes, and not for individual risk assessment^3^.

The main source for the evaluation of neutron radiation weighting factors is experimental RBE data from *in vitro* cell-killing, chromosome aberration studies and animal experiments. A description of experimental data concerning neutron RBE can be found in BEIR VII^4^ and references therein. As an obvious consequence of the fact that the secondary particle field induced by neutrons varies with neutron energy, also neutron RBE depends on neutron energy, and this has been an important ingredient of successive review and corrections of adopted radiation protection standards over time. The most significant change in neutron w_R_ from ICRP Publication 60^5^ to its reevaluation in ICRP Publication 92^6^ stemmed indeed from the disregard of the much higher photon component of the neutron dose when low energy neutrons interact in a larger size receptor. Caution is therefore needed, when trying to extrapolate results for small receptors as mice to humans. More in general, radiation protection recommendations from international commissions are liable to change over time, following the essential efforts of the commissions to revise and include newly produced data. As a further example, an additional reduction of w_R_ for high energy neutrons (above 1 GeV) has also been introduced, from ICRP Publication 92 to ICRP Publication 103^3^.

Another interesting lesson can be drawn when comparing the currently adopted ICRP^3^ and U.S.NRC standards^7^ for neutron weighting factors (referred to as quality factors in the US standard): despite the agreement on the maximal effectiveness for 1 MeV neutrons, the magnitude of this enhancement is different (established values are differing by a factor of two), and an additional increase in effectiveness is considered at energies around 20 MeV by U.S.NRC only. The established radiation protection standards therefore depend on the dataset adopted for the evaluation^8^.

New experimental and theoretical efforts have been recently undertaken in the framework of the European project ANDANTE^9^, with the overarching objective of determining values of RBE for neutrons for specific tissues and neutron energies. The context of the project is specifically the possible role of secondary neutrons in the induction of second primary neoplasms following particle therapy, especially for pediatric patients. It is clear that this issue demands a renewed and great attention to unravel mechanisms behind neutron biological effectiveness and its energy dependence.

It is also worthwhile to notice that, reviewing the literature, non-targeted effects in cellular responses to neutrons are debated, and no conclusive evidence exists to support or refute their existence^10^,^11^. However, once again, the strong dependence of energy depositions in the target on neutron field characteristics and target geometry clearly implies that no results on biological effectiveness or neutron induced bystander effects can be given unambiguously without a full description of the radiation field at the point of interest.

In the light of all this, a theoretical effort to trace back neutron biological effectiveness to first principles (as much as this is possible) seems highly desirable. In this work we couple the potential of two different modeling approaches, namely radiation transport and track structure calculations, in order to evaluate the biological effects of neutron exposures of tissue targets at cellular/sub-cellular level. The Monte Carlo code PHITS^12^ is used to properly characterize on a microscopic scale the secondary charged particle field emerging from neutron interactions as a function of neutron energy and position in the receptor. Gathered information on the secondary particles are condensed into the most relevant quantities to be fed into the Monte Carlo code PARTRAC^13^,^14^, which finally delivers the pattern of radiation induced cellular damages to nuclear DNA associated to the neutron field. Albeit not exhaustive of cellular endpoints, DNA damage is here chosen as a good indicator of radiation clustering properties and therefore of radiation quality. In particular, clustered DNA lesions, *i*.*e*. clusters of two or more lesions within few helical turns of the DNA, are used to quantify neutron effectiveness, their biological importance being well recognized within the radiation research community^15^,^16^,^17^.

When neutron effects are compared to a chosen reference radiation field, the established simulation framework allows the evaluation of different energy dependent neutron RBE models. To our knowledge few RBE models for neutrons exist all together, from older applications to neutron therapy^18^,^19^ up to recent neutron RBE for DNA damage induction^20^, and the one here presented for clustered DNA damage induction is the first fully based on *ab-initio* calculations starting from physical interactions.

## Methods

### Neutron Transport Calculations with PHITS

The PHITS (Particle and Heavy Ion Transport code System, v. 2.52) code has been used to simulate the exposure of an ICRU44 soft tissue^21^ spherical phantom (ICRU sphere geometry) immersed in an isotropic field of monoenergetic neutrons. PHITS is a multi-purpose Monte Carlo code dealing with particle transport (via continuous energy loss), collisions and decays (via reaction models and cross section data libraries). For neutron induced reactions below 20 MeV, PHITS was run in the so-called Event Generator Mode, thus delivering information on an event-by-event basis using the cross sections from Evaluated Nuclear Data libraries. For high energy neutrons (and other particles), the JAM4 and JQMD5 models are implemented to simulate particle induced reactions up to 200 GeV and the nucleus-nucleus collisions, respectively. Full details on the code can be found elsewhere^12^.

The spherical phantom has radius R = 15 cm, intended to roughly reproduce the size of a human trunk^22^,^23^. We consider three different inner scoring regions with spherical shape and with equal radii r = 1.5 cm, but with centers positioned at different distances d with respect to the phantom center along a common diameter: the most internal scoring region is concentric to the phantom (d = 0 cm), the mid-depth one is at d = 7.5 cm and the most external is at d = 13.5 cm, touching the surface of the phantom. All quantities extracted from transport calculations are always given as a function of primary neutron energy, irrespective of how such energy is modified during the interactions in the phantom.

A complete set of information on the mixed field of secondary particles generated by neutron interactions in the scoring regions can be obtained with PHITS, as *e*.*g*. their energy differential fluence spectra, energy deposition, relative abundances, Linear Energy Transfer (LET) spectra, *etc*. In transport calculation such quantities are typically obtained on a macroscopic scale: the linear dimension of the three scoring regions in our simulation setup is 3 cm, and the computational expense of the simulation necessarily increases when going to smaller scoring regions, because of the reduced probability of energy deposition per source particle in a smaller volume. For the purpose of this work, a characterization of the radiation field at the cellular (and sub-cellular) scale is necessary: our aim is to use results of the transport code as input to the track structure model, where a single cell model is implemented as target of radiation action (see the section on PARTRAC for details). With this aim, our strategy was to first understand if the wealth of simulated data on the secondary particle field could be condensed in such a way that only few quantities, the most relevant for biological effects, could be fed into the track structure code. This is also a potential advantage if we want to establish a calculation framework which can be easily extended to other works and/or Monte Carlo codes.

To achieve all these objectives, we propose an approach based on:

- the use of the microdosimetric functions implemented in PHITS^24^,^25^, which output probability densities of microdosimetric quantities in macroscopic matter. Such functions allow for a proper characterization of the stochastic energy deposition by radiation at the cellular and sub-cellular scale of interest, without increasing the computational time. In particular, the frequency f(y) and dose distribution d(y) of the lineal energy y are used. The lineal energy is defined as the ratio of the deposited energy in a sensitive site to the mean chord length of the site^26^,^27^. Distributions f(y) and d(y) give respectively the probability to find or the dose deposited by particles with a lineal energy between y and y + dy in the sensitive site;
- the simplifying assumption that the whole amount of data on the mixed field generated by neutron interactions can be condensed in two representative pieces of information for each of the secondary charged species, namely: their relative contribution to the total neutron dose; and a single indicator of their clustering properties in terms of energy transfer per path length, which is known to be correlated to radiation induced cellular effects^1^. This latter (as it will be described later in detail) is the first moment of the d(y) distribution, referred to as the dose-mean lineal energy ȳ_D._ The sensitive site is chosen to be a sphere with diameter 1 μm, roughly corresponding to the linear dimensions of chromosome domains. The dose-mean lineal energy in target sizes of 1 μm (or smaller) is assumed to be correlated to biological effectiveness for many cellular endpoints^28^,^29^,^30^. Only these two parameters per secondary species are needed from transport calculations to fully deduce neutron biological effectiveness as a function of their energy. Even if not explicitly taken into account, ȳ_D_ values for all species together with their dose contributions also enter in the definition of a single ȳ_D,n_ for the neutron field. This will also be given and used to quantify neutron effectiveness in a first reference calculation.

PHITS microdosimetric functions have been tested comparing results for ȳ_D_ to results for the dose average LET values calculated in the corresponding macroscopic regions: despite the difference in the definition of the two quantities, dose averaging yields compatible numerical values. This leads to the conclusion that the dose-mean lineal energy extracted from PHITS is representative of linear energy transfer averaged over the nuclear volume, which will be extracted from PARTRAC (see the following section).

PHITS simulations were run for primary neutron energies in the range 10^−5^–10^3 ^MeV, covering the typical energy range in which radiation protection factors are given. Going to even lower energies, down to thermal neutrons, we verified that no significant differences can be found in terms of the secondary charged particle induced field, both for the relative dose contributions of secondary charged species and for their linear energy transfer on a micrometric scale. Theoretical arguments supporting this finding are given in the Results section. This has also been verified by calculations not included in this work. Results are always obtained with a statistics of at least ten million neutrons per run (10^4^ neutrons per batch per 10^3^ simulation batches), and averaged over up to 5 runs when energy deposits are low (for E_n_ ≤ 0.1 MeV), in order to reduce statistical fluctuations. Calculations can be performed on a standard PC (2.2 GHz in our case), with a maximal duration of 48 hours per run. Errors on quantities from a single run are standard deviations among results for each batch, as given by the code. Errors in case of run repetitions are standard deviations among results for different runs. In this way, relative errors are always kept under few per cent (with errors up to tens of per cent only when the dose contribution of a specific type of particle is very low, *i*.*e*. the corresponding reaction channel has a very low cross section). Simulations for the reference photon field for the evaluation of neutron RBEs were run with the same setup, scoring field characteristics in the most external scoring region. The spectrum of X-rays generated by a Xstrahl-200 machine has been used (220 kV field, 2 mm Cu filter)^9^.

### Track Structure calculations with PARTRAC

PARTRAC^13^,^14^ is a well established biophysical Monte Carlo code allowing the simulation of radiation track structure up to the evaluation of different types of cellular damage as a function of radiation quality (photons and charged particles) and dose. In particular, physico-chemical processes leading to DNA damage (direct and indirect, through radicals) can be simulated when particles traverse the cell model implemented in the code, which contains a realistic description of the human genome. The PARTRAC code was recently upgraded in order to properly deal with interaction cross sections for full slowing down ions^31^.

In this work, the sensitive target for secondary charged particles accelerated by neutrons is chosen to be the whole genome of a human fibroblast in its G_0_/G_1_ state, with levels of organization ranging from DNA double helix in atomic resolution up to chromosomes. The cellular nucleus is modeled as an ellipsoid with axes of 20, 10.6 and 5.4 μm respectively along the x, y and z axes. The cytoplasmic compartment is a rectangular box containing the nucleus. Simulations of single-cell irradiation were performed for secondary charged species found in the mixed field, in a wide range of initial energy (0.0625–64 MeV/u). Calculations were run with two different geometries: in the first case, when the initial energy is such that the particle can traverse the cell nucleus in all its thickness along z, the source region is a grid of (5 × 3) pixels, each of which has a surface of 16 μm^2^. The grid is positioned at a fixed distance of 0.3 μm along the z-axis below the nuclear surface. In each of the simulation run, a particle is emitted upwards from a random point in each of the pixel, so that the overall particle fluence is of 0.0625 particles/μm^2^. As for the estimator of linear energy, the linear energy transfer (LET) over the microscopic nuclear volume can be derived from the equation:
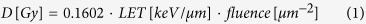
where D is calculated from the scored energy deposit in the cell nucleus in the simulations. In the second case, when the energy is too low and the particles would stop in the nucleus on their path along z, we have calculated DNA damage resulting from particles emitted isotropically with starting points randomly distributed in the cellular volume (at a fixed starting point density of 0.008 μm^−3^). In this case equation (1) does not provide a useful indicator of the linear energy transfer, and a better one is simply given by the initial energy of the particle divided by its track length. It is worth noting that linear energy transfer indicators obtained from track structure calculations are different in their definition from the dose-mean lineal energy derived for secondary species in transport calculations, even if the paths travelled in the nucleus are of few μm. However, as mentioned, dose averaging evens out such difference, and ȳ_D_ values from PHITS can be matched to LET values from PARTRAC.

Cell irradiations with the chosen reference photon field for the evaluation of neutron RBEs were also simulated. The spectrum of X-rays generated by a Xstrahl-200 machine has been used (220 kV field, 2 mm Cu filter)^9^.

Results on DNA damage presented in this work are always obtained with a statistics of at least 64 runs and given as averages for a dose delivery of 1 Gy per cell nucleus and as a function of particle LET. Calculations were performed in parallel for 12 runs on our local 12 2.4 GHz CPU server, with a maximal duration of 72 hours per 12 runs, depending on input parameters for ion energy and starting points. We score both the overall yield of DNA fragments shorter than 30 bp (complex lesions^23^) and DSB clusters, namely the number of lesions containing at least two DSBs within a distance shorter than 25 bp^14^,^32^. Errors on the yields of short DNA fragments are calculated assuming a Poisson counting for the overall statistics. Errors on DSB cluster yields per run are obtained as standard deviations among results for different runs. The standard deviations among doses to the nucleus in different runs are also calculated, and error propagation is used to account for variations in the damage yield per Gy.

## Results

### A phenomenological indicator of biological effectiveness for a mixed field

**Figure 1:**
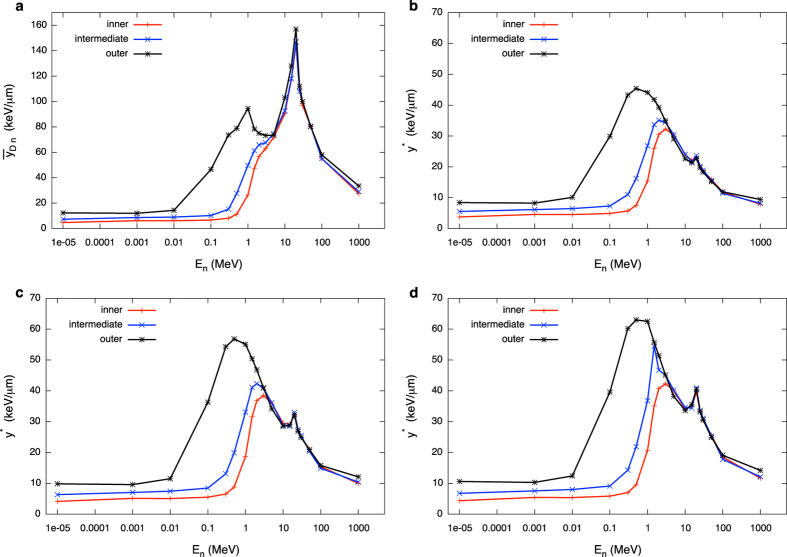
Variation with neutron energy and location in the phantom of neutron dose-mean lineal energy and saturation corrected dose mean lineal energy. Dose-mean lineal energy (**a**) and saturation-corrected dose-mean lineal energy for saturation parameter y_0_ = 100 (**b**), 150 (**c**) and 200 keV/μm (**d**) for neutrons as a function of their initial energy in the three scoring regions in the phantom. Error bars are standard deviations among different PHITS runs and are always within the symbols. Lines are drawn to guide the eye.

In this section we start using PHITS to quantify neutron effectiveness based on lineal energy without recurring to an explicit characterization of the neutron induced mixed field. This will serve as reference, once results on the secondary charged particle field are introduced. The value of the lineal energy for indirectly ionizing radiation is derived from energy depositions of the secondary charged products. Information carried by the dose distribution d(y) can be condensed in the first moment of the distribution, referred to as the dose-mean lineal energy ȳ_D_:
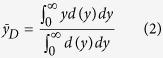


The ȳ_D_ quantity is the lineal energy with which, on average, the dose is delivered by radiation in the site of interest. If we forget about the variety of species accelerated in the mixed field, a single value of ȳ_D_ can be defined. Such value depends on the dose distributions of the lineal energy for all species in the field and on their relative contribution to the total dose. The global d(y) distribution necessarily possesses a complex structure, potentially with Bragg peaks at different y values arising from particles of different types stopping in the scoring volume. The first moment of d(y) loses therefore its power of condensing the information carried by the overall distribution. Results for neutron ȳ_D,n_ as a function of their initial energy in our modeling setup are shown in Fig. 1a for the three scoring regions in the spherical phantom. For the most external region (*outer*), where the actual neutron energy is close to the nominal primary energy value, a double peaked structure is observed: ȳ_D,n_ reaches maximal values with respect to neighboring energies for neutrons of about 1 and 20 MeV. For deeper regions in the phantom (*intermediate* and *inner*), lower energy neutrons are easily moderated and the resulting lineal energy of the mixed field is decreased. In the higher neutron energy range, ȳ_D,n_ reaches values higher than 100 keV/μm (Fig. 1a). Such high values can be reached only by low-energy ions with Z > 2^33^,^34^, which means that slow recoiling nuclei or nuclei produced in nuclear reactions have an important weight in determining the overall dose-mean lineal energy. However, at such high linear energy transfer, a decrease in biological effectiveness can be measured for survival-related endpoints: a lowest fraction of cells survives when hit by high LET radiation, and the risk per irradiated cell therefore decreases (overkill effect). Also, a smaller number of cells is affected by radiation per unit dose, since a larger energy is deposited to hit cells, and averaging across the whole cell population is misleading. This can be taken into account, at least phenomenologically, introducing the saturation-corrected dose-mean lineal energy y*^28^,^35^:
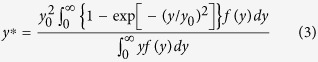
where the saturation parameter y_0_ can be fixed to a value equal or higher than 100 keV/μm, thus reducing the weight of higher y component in the field. The quantity y* can be used as an indicator of biological effectiveness. Results for y* as a function of neutron energy and position in the phantom are given in Fig. 1b–d, for y_0_ = 100, 150 and 200 keV/μm. As a consequence of the saturation correction, the magnitude of the increase of lineal energy for neutron energies around 20 MeV is largely reduced, the more the higher is the chosen value for the saturation parameter. The position of the peak of maximal effectiveness is found at 1 MeV when such nominal neutron energy corresponds to the actual one in the scoring region (*outer*), and it is shifted to higher energies for deeper-seated targets.

### Reaction mechanism interplay in determining secondary particle contributions to the neutron dose

The first piece of information to be extracted from neutron transport calculations in order to fully characterize the secondary particle field is the relative contribution of accelerated species to the total neutron dose. In our calculations, only secondary charged species are considered: the photon component of the neutron dose is scored as energy depositions of the tertiary electrons accelerated by the photons. Results on the secondary particle contributions to the neutron dose are discussed in this section with explicit reference only to the dominant reaction mechanisms. A detailed discussion of the wealth of possible reactions is beyond the scope of this work.

**Figure 2:**
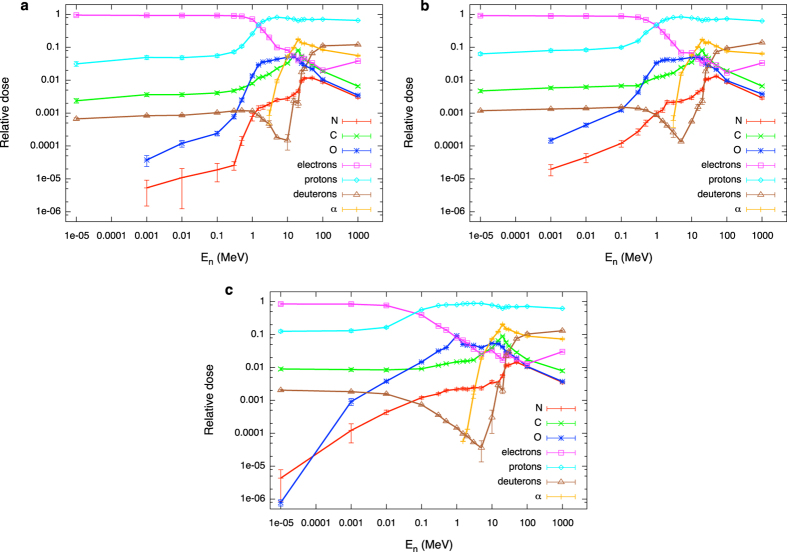
Variation with neutron energy and location in the phantom of the relative contribution of secondary charged species to the total neutron dose. Relative contributions of secondary charged species to the total neutron dose as a function of neutron initial energy: (**a**) inner; (**b**) intermediate; (**c**) outer scoring region. Error bars are standard deviations among results for batches in a single run as given by PHITS. Lines are drawn to guide the eye.

For a given particle species, the total dose can be easily computed as the integral of the dose distribution of the lineal energy d(y_s_) over the explored lineal energy range. The relative contribution is obtained further normalizing such integral to the corresponding one for the overall neutron d(y_n_). In Fig. 2 this is shown as a function of neutron energy and position in the phantom for C, N, O nuclei, electrons, protons (H nuclei), deuterons and α particles. Not included in the analysis are species (tissue elements) with atomic number Z > 8, whose contribution is very low: only for the highest neutron energies such species contribute up to few per cent to the total dose (*e*.*g*. ~3.5% for neutron energy of 100 MeV). The highest energy range for neutrons might deserve a dedicated investigation also for the possible induction of other reaction process (as π/EM cascades), when studying the effects of accelerated neutrons in the space radiation environment^36^,^37^.

Over the whole neutron energy range the largest fraction of deposited dose is either due to electrons arising from photon interactions or due to secondary protons. When dominant, the electron component stems from the 2.2 MeV photons emerging from neutron capture processes on target H nuclei. A deuteron is formed as a result of the capture, and a photon is emitted carrying the energy gained in binding (p(n, γ)d in the common notation of nuclear reactions). Such photons further accelerate electrons, which are responsible of energy deposition to the target. Neutron capture cross section on target protons decreases with increasing neutron energy, as it is evident in the drop of the electron dose. The primary neutron energy at which this happens depends on the depth of the scoring region in the phantom, and it is lower for the most external scoring region (panel c), when only part of the neutrons has the chance to be moderated down to energies where the capture process is always dominant. Deuterons are the recoiling products of neutron capture reactions: their dose contribution is several orders of magnitude lower, and drops down in the same way as the electron component. After the drop, new reaction channels open with increasing neutron energy, either photons (electron dose) or deuterons can be newly produced, and the corresponding dose contributions rise again (see in the following for details on possible reaction mechanisms at higher neutron energies). Always at low neutron energies, when the electron component of the neutron dose is dominant, the small proton component is largely due to ^14^N(n,p)^14^C capture processes: the proton and the carbon nucleus are accelerated sharing the gain in energy of 626 keV. At neutron energies below 10^−5 ^MeV down to thermal neutrons, no significant differences are expected for the induced secondary charged particle field: no-threshold neutron capture reactions on target H and N nuclei are largely dominant, the cross sections for such reactions are evaluated to increase with decreasing neutron energy in such a way that their ratio is constant, and their relative dose contribution to the total neutron dose will also stay constant.

At higher neutron energies, recoiling protons largely dominate the neutron dose: out of nuclei present in the traversed tissue protons (H nuclei) can acquire the maximal energy in a single collision with a neutron, since the maximal energy transfer is given by:
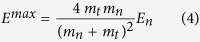
where E_n_ stands for the incoming neutron energy and m_n_ and m_t_ are the neutron mass and the target nucleus mass, respectively. Heavier tissue elements also contribute to energy deposition, their weight becoming higher as neutron energy increases. The importance of different heavier species is governed by several factors:

- their relative abundance in the tissue, since more abundant species are most likely to be hit by a neutron and acquire recoil energy;
- the cross sections for inelastic collisions with neutrons. An inelastic collision leaves the target in an excited state, and decay products will further deposit their energy;
- the onset of nuclear reactions at neutron energies higher than thresholds dictated by energy conservation for each possible target (*e*.*g*. approximately 10, 1 and 5 MeV for collisions with C, N, O targets^38^). As an example of such reactions, the neutron might be absorbed by the target nucleus, and the fused system will have enough energy available (from the kinetic energy of the neutron and from the gain in binding energy) to emit photons or particles. In alternative, the hit target might fragment without any intermediate fused system being formed. Generally speaking, the specific outcome of a nuclear reaction is determined by reaction channel branching ratios, obeying to conservation and statistical laws. As a result, fragments with charge different than the reaction target can be produced, as *e*.*g*. α particles. As can be seen from Fig. 2, the α component of the dose appears only as the neutron energy increases in our calculations. The energy at which this happens is specifically determined by energy thresholds for possible (n, α) reactions on different tissue targets.

As mentioned, the actual energy dependence of neutron induced reactions is masked when scoring regions deep inside the phantom are considered (panels a and b in Fig. 2), as neutrons reaching such targets might have lost part of their energy in a previous interaction. This is also dependent on neutron energy, and it is true in particular for those energies at which the neutron mean free path is shorter than the path neutrons have to travel in tissue to reach the scoring volume.

### A detailed study of energy depositions of secondary species

**Figure 3:**
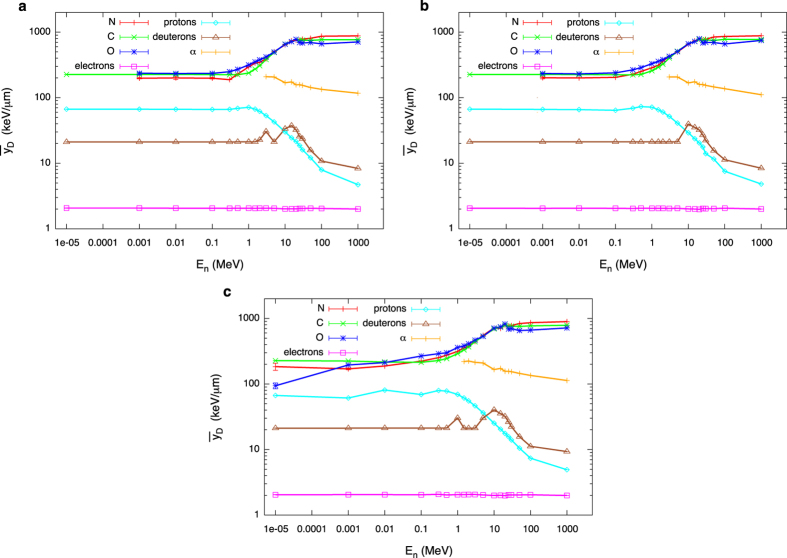
Variation with neutron energy and location in the phantom of the dose mean lineal energy of secondary charged species. Dose-mean lineal energy values for secondary charged species accelerated by neutrons as a function of neutron initial energy: (**a**) inner; (**b**) intermediate; (**c**) outer scoring region. Error bars are standard deviations among different PHITS runs and are in most cases within the symbols. Lines are drawn to guide the eye.

The dose distribution of the lineal energy can be calculated for each of the secondary charged species present in the mixed field. The sum of such components, weighted by the relative dose contribution, gives the overall neutron d(y_n_). A dose-mean lineal energy can be attributed to each species s: the first moment ȳ_D,s_ is an excellent parameter to characterize a single peak lineal energy distribution d(y_s_). Results for ȳ_D,s_ are shown as a function of neutron energy and position in the phantom in Fig. 3 for all considered secondary species.

As it is evident at a first glance, for neutron energies below around E_n_ = 1 MeV, secondary protons are characterized by a high and almost constant value of ȳ_D_. Such value stays high up to around 1 MeV neutron energy, and then decreases as the neutron energy further increases. In the lower neutron energy range however, the dose contribution by protons is very low, hence such a high value of the proton lineal energy does not lead to a high overall ȳ_D,n_. When the proton dose contribution increases, ȳ_D,n_ also gets to a higher value, before starting to decrease again, this time because of the decrease in proton lineal energy itself (*i*.*e*. the acceleration of higher energy protons). This is the origin of the first peak of the overall neutron ȳ_D,n_ reported in Fig. 1a as a function of neutron energy^23^. The second peak observable in the overall lineal energy of the mixed field can be attributed to the rapid increase of ȳ_D_ for heavier nuclei (C, N, O), together with their increasing weight in the neutron dose for neutron energies above 1 MeV. Electrons are always characterized by a constant and rather low dose-mean lineal energy, independently on their mechanism of production. Deuterons have a constant and low ȳ_D_ when they are the recoiling products of neutron capture reactions on H nuclei (for low neutron energies), while they can explore a wider range of lineal energies and reach higher ȳ_D_ values when produced via other reaction mechanisms, given the wider energy range in which they can be accelerated and possibly stopped in the target. The α component is present only for neutron energies above their production thresholds. Emitted α particles have, from their appearance on, a decreasing ȳ_D_, as more energy is available in the reaction when the neutron energy increases and they can be accelerated to higher kinetic energies (*i*.*e*. lower stopping power and hence lower lineal energy values).

### DNA damage calculations as a function of linear energy transfer

Results of DNA damage obtained with PARTRAC for charged species are given as damage yield per unit Gy per cell as a function of linear energy transfer. Damage is scored both in terms of the yield of DNA fragments shorter than 30 bp and of DSB clusters (see Methods section for the definition).

**Figure 4:**
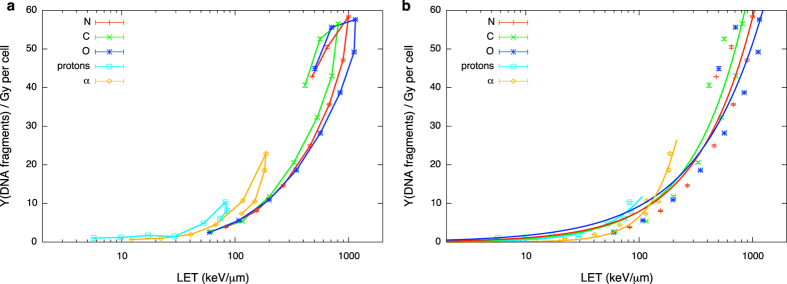
Charged particle induced DNA fragments as a function of LET. Yield of DNA fragments shorter than 30 bp per Gy per cell as a function of LET for different charged particle species. Lines are drawn to guide the eye in panel (**a**), while analytical fit functions (equation (5)) are plotted in panel (**b**). Error bars are given according to a Poisson counting of the fragment yield for the whole statistics, taking into account propagation with the standard deviation of the dose to the nucleus among different PARTRAC runs.

In Fig. 4a we show the yield of DNA fragments shorter than 30 bp. The number of fragments (a possible measure of the complexity of DNA damage) becomes higher as the LET increases (or, equivalently, the initial energy goes down). Different ions at the same LET induce damage of different complexity, which is related to the energy of secondary electrons: from the Z^2^/β^2^ dependence of LET (where β is the ratio of particle velocity to the speed of light), the same linear energy transfer for ions of different charge implies that the heavier one has a higher velocity. The velocity of secondary electrons is mainly determined by the velocity of the primary ions; faster ions produce secondary electrons of higher energies. Therefore, when comparing ions of different charge but at the same LET, the faster one undergoes fewer interactions per unit track length, but with a higher energy expense per interaction. This increase in the ion mean free path results in a reduced probability of damage clustering. After the maximum LET value, particle tracks become shorter enough to be fully contained in the cell nucleus and the corresponding damage is found to be lower. Despite having the same LET, two particles at the proximal and distal edge of the Bragg peak have different velocity, they might have different charge state (charge pick-up processes come at play for slow ions) and they therefore have very different track structures. Differences in radial dose distributions, mean free path of interactions and energy deposit per interaction again translate into different complexity of the DNA damage. As a consequence, lines connecting simulation points show hooks, and, at least for heavier ions (C,N,O), a fragment yield cannot be unambiguously associated to a given LET value. In principle, as it will be discussed later, this prevents the coupling to neutron transport based on a linear energy transfer indicator. However, analytic functions approximating the yield of fragments as a function of LET can be given for each species, even if this necessarily introduces an error associated to the neglected dependence of such yield on particle energy, in particular for the heavier nuclei. In this work, fragment yields have been fitted by:
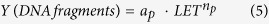
where a_p_ and n_p_ are fit parameters depending on particle type. Best fit curves are shown in Fig. 4b.

**Figure 5:**
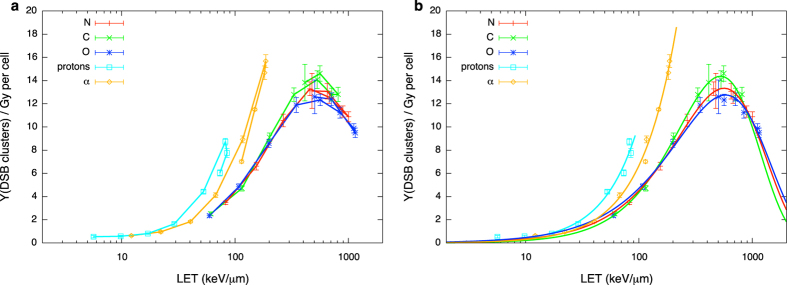
Charged particle induced DSB clusters as a function of LET. Yield of DSB cluster (as defined in the text) per Gy per cell as a function of LET for different charged particle species. Lines are drawn to guide the eye in panel (**a**), while analytical best fit functions (equation (6)) are plotted in panel (**b**). Error bars are given taking into account propagation of standard deviations among different PARTRAC runs for DSB cluster yield and dose to the nucleus.

In Fig. 5a we show the yield of DSB clusters as a function of LET. At difference with the total number of fragments, the yield of clusters include the information on the spatial distribution of damage: following the definition, a cluster contains a minimum of two DSBs in a distance shorter than 25 bp, which gives a single DNA fragment shorter than 30 bp, up to many DSBs and a corresponding high number of fragments when it is induced by a densely ionizing ion track. For light ions results closely follow the trend of the fragment yield, with average cluster multiplicity (*i*.*e*. number of DSBs in the lesion) close to 2 for protons and α’s. For heavier nuclei instead, the number of clusters starts decreasing before the maximum LET is reached. As we understand comparing Figs 4a and 5a, when the energy becomes lower the damage becomes more spatially localized. The interesting feature we observe in DSB clusters vs. LET trends is that a damage can now be associated to a single LET value without (or, rather, with a reduced) ambiguity related to the particle energy under consideration. This means that differences in the complexity of damage induced by two particles with the same LET but at the proximal and distal sides of the Bragg peak are hidden. As a consequence, simulation points can be very well fitted with analytical functions, once a factor is introduced for heavy ions to take into account damage clustering:
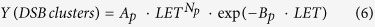
where A_p_, N_p_ and B_p_ are fit parameters depending on particle type, while the simple power law dependence of equation (5) (two parameter fit) can still be used for proton and α induced damage. Best fit curves are shown in Fig. 5b.

### Coupling of transport and track structure calculations

**Figure 6:**
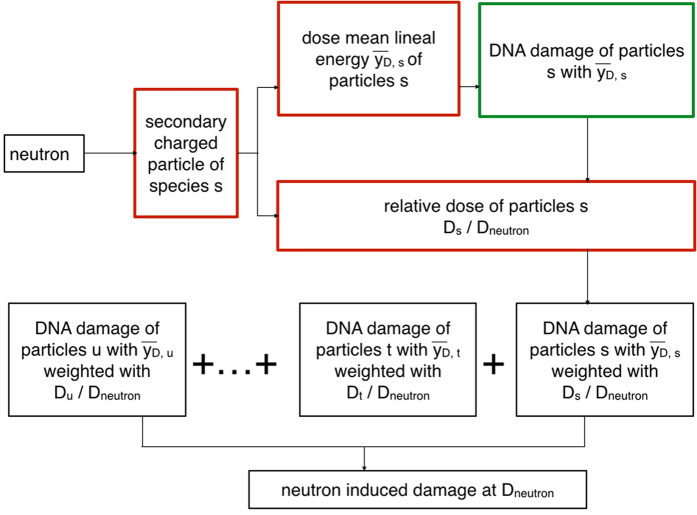
Proposed coupling scheme for transport and track structure results. Calculation scheme followed for the coupling of transport (PHITS) and track structure calculations (PARTRAC) to obtain neutron induced DNA damage. Red boxes identify information from PHITS, green boxes information from PARTRAC. Details are given in the text.

In order to couple the results on the secondary charged particle field induced by neutrons to DNA damage evaluated by means of track structure calculations, we follow the calculation scheme of Fig. 6: once that a dose-mean lineal energy ȳ_D s_ is associated to a given species *s* in the secondary field, the type of damage under investigation induced by *s* particles per unit dose per cell at the same linear energy transfer is scored using best fit analytical functions from equation (5) or (6). This damage is further weighted with the relative dose contribution of *s*: this converts the dose of 1 Gy into the corresponding fraction of the neutron dose delivered by secondary *s* particles. The damage is therefore now given as *s* - induced damage per unit neutron dose per cell. When this is done for all secondary species, damages can be summed up thus obtaining the overall neutron induced DNA damage per unit dose per cell. Deuteron induced damage as a function of LET is the same as for protons. Electron damage at their almost constant LET is also calculated with the proton curve at the same LET value (highest energy protons). Errors on final damage yields are obtained taking into account errors on relative dose contributions of secondary species as calculated by PHITS.

**Figure 7:**
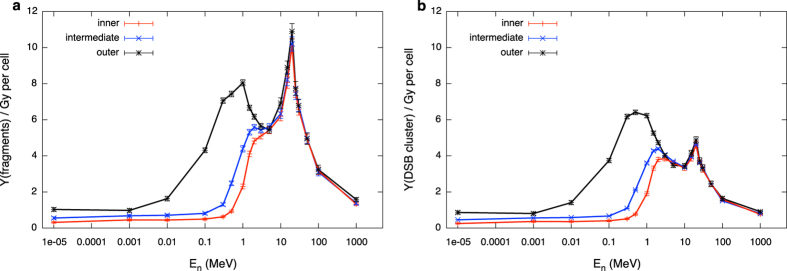
Variation with neutron energy and location in the phantom of neutron induced DNA damage. Neutron induced DNA damage: (**a**) DNA fragments shorter than 30 bp; (**b**) DSB clusters; damage per Gy per cell as a function of neutron initial energy in the three scoring regions in the phantom. Lines are drawn to guide the eye. Error bars come from standard deviations among results on doses of secondary species for batches in a single run as given by PHITS.

Neutron induced DNA fragment production and DSB cluster induction are given respectively in the two panels of Fig. 7 as a function of neutron energy and for the different scoring regions. Predictions on neutron induced DNA fragment yields are necessarily affected by the fact that equation (5) does not offer a fully suitable replacement for simulated data of charged particle induced DNA fragment yields vs. LET, as shown in Fig. 4. Conversely, looking at Fig. 5, we can conclude that the approximation introduced by the use of equation (6) does not have a significant impact on the predictions of neutron induced DSB cluster yields. In both cases, two regions of maximal effectiveness in damage induction are found, for neutron energies of about 1 and 20 MeV, respectively.

Slow secondary protons are mostly responsible for the maximal effectiveness of neutrons at energies around 1 MeV^23^. On the contrary, damage induction by heavy nuclei dominates the effectiveness in the second peak, for E_n_ ~ 20 MeV. When switching from fragment yield to DSB cluster as endpoint (hence when spatial clustering of damage comes at play), the height of the second peak is greatly reduced. This illustrates that the overall shape (peaks, heights) of neutron effectiveness depends on the type of damage under consideration, and how that evolves with LET.

The dependence on where the secondary field is evaluated (hence on the receptor geometry) also emerges clearly from the comparison of data for the three scoring regions. For the outer volume (low neutron moderation) neutron energy in the volume is closer to nominal neutron starting energy, which means that the corresponding damage can be associated to a local evaluation of neutron energy throughout an exposed target.

### Evaluation of neutron RBE from saturation-corrected dose-mean lineal energies and DSB cluster induction

As mentioned, for the radiation quality under investigation, a RBE (Relative Biological Effectiveness) value is conventionally defined as the ratio of the dose of a reference radiation to the dose of the test radiation inducing the same effect. RBE values are therefore a measure of effectiveness related to a specific biological endpoint.

In the simulation framework established in this work, neutron RBE values can be obtained with different sets of results. We present in this section neutron RBE values obtained from saturation-corrected dose-mean lineal energies and from DSB cluster induction. For the evaluation of RBE we always use quantities calculated for the most external scoring region of the tissue phantom, where neutron energy in the region is closer to nominal neutron starting energy for an external irradiation. Neutron RBE results for the most internal and mid-depth scoring regions as a function of primary neutron energy are included as Supplementary Material. For the reference photon field we have used the X-ray spectrum generated by a Xstrahl-200 machine (220 kV field, 2 mm Cu filter)^9^.

**Figure 8:**
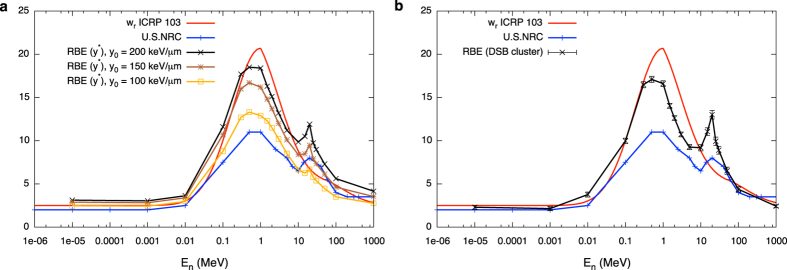
Model results for neutron RBE as a function of their energy. Neutron RBE as a function of energy evaluated from: (**a**) ratio of saturation-corrected dose-mean lineal energies with y_0_ = 100 (yellow squares), 150 (brown asterisks) and 200 keV/μm (black crosses); (**b**) DSB cluster induction (black crosses). Lines are drawn to guide the eye. The reference photon field is the X-ray spectrum generated by a Xstrahl-200 machine (220 kV field, 2 mm Cu filter). Results are given for the more external scoring region of the phantom. For a qualitative comparison, ICRP (in red, continuous function) and U.S. NRC (blue points connected by lines) standards for weighting factors are also plotted in both panels. Error bars on RBE from saturation-corrected dose-mean lineal energies come from standard deviations among different PHITS runs for neutron and X-ray y* values, and are within symbols. Error bars on RBE on from DSB cluster induction come from errors on the DSB cluster yields for neutrons and X-rays.

The saturation-corrected dose-mean lineal energy introduced with equation (3) has been frequently used as an indicator of biological effectiveness. In this work, when using transport calculations only, the phenomenological saturation correction allows us to neglect explicit consideration of the neutron induced charged particle field. If we assume a correlation between such indicator for neutrons relative to photons and the corresponding enhancement in biological effect, the RBE is simply given by the ratio of neutron y* to the corresponding saturation-corrected dose-mean lineal energy value for photon irradiation. This can be done at all neutron energies, then obtaining an energy dependent neutron RBE model. Results shown in Fig. 8a are obtained dividing y* of Fig. 1 (panels b, c and d for different choices of the y_0_ value) to the y* value obtained in the photon irradiation of the phantom simulated with PHITS.

Thanks to the approach for coupling transport and track structure calculations proposed in this work, a neutron RBE model can also be obtained using DNA damage induction: PARTRAC easily allows the evaluation of photon induced DNA damage. Under the assumption of the linearity of the chosen DNA damage endpoint with dose, neutron RBE can be extracted from the evolution of the measured endpoint as a function of neutron energy, divided by a measure of the endpoint following exposure to the photon reference field. This is assumed to be true for the DSB cluster endpoint, since the probability of two particle tracks cooperating in inducing damage on such a short genomic length is very low, and the final yield of DSB clusters ultimately depend on the number of tracks traversing the cell nucleus, which is in turn linearly correlated to the dose. The energy dependent neutron RBE for DSB cluster induction can therefore be obtained as the ratio of the yield of clusters following neutron irradiation to the yield of clusters following photon irradiations. This is shown in Fig. 8b.

The substantial agreement between the mechanistic model based on clustered DNA damage induction and the phenomenological model based on y* (when an appropriate value for y_0_ is chosen) can be seen as offering a justification and independent validation of this latter. This is further discussed in the next section. For both approaches, obtained RBE values are plotted together with current standards for radiation weighting factors for a qualitative comparison, as also commented in detail in the Discussion section.

## Discussion

An *ab-initio* approach to trace back the origin of neutron biological effectiveness as a function of energy is presented in this paper. The coupling between neutron transport calculations performed with PHITS and track structure calculations up to DNA damage induction performed with PARTRAC delivers the pattern of neutron induced DNA damage as a function of energy and at different depths in an ICRU44 spherical tissue phantom immersed in an isotropic monoenergetic neutron field.

At first, the correlation between the evolution of neutron dose-mean lineal energy and their effectiveness as a function of energy is explored, without explicitly taking into account the mixed nature of the secondary particle field and without recurring to track structure modeling. Neutron ȳ_D,n_ has a maximum around 1 MeV, where we expect neutron maximal effectiveness from RBE data and weighting factor standards, but then it becomes even higher for higher neutron energies, because of the important contribution in dose and corresponding weight for lineal energy of heavy nuclei as C, N, O in the secondary field (Fig. 1a). A phenomenological saturation correction with saturation parameter y_0_ in the range 100–200 keV/μm can be applied to lineal energy distributions in order to take into account the so-called overkilling effect in survival-related RBE, thus obtaining the so-called saturation-corrected dose-mean lineal energy (Fig. 1b–d), which can be used as an indicator of biological effectiveness and which will be further used to extract a possible RBE model.

The secondary charged particle field induced by neutrons is then characterized in terms of relative dose contributions of different species to the total neutron dose (Fig. 2) and their corresponding dose-mean lineal energies (Fig. 3). We discuss in detail how such quantities vary as a function of neutron energy and location in the receptor, which can be traced back to the interplay of different reaction mechanism (neutron cross sections) and chances of neutron moderation in the target.

DNA damage evolution with linear energy transfer is predicted with PARTRAC and it is found to vary for different types of damages (as short DNA fragments or DSB clusters) and for different radiation qualities. For the considered species, the yield of short DNA fragments keeps increasing up the maximal LET and then it is decreased for particles below their maximal stopping power (see Fig. 4). On the contrary, when spatial distribution of DNA damage is taken into account, the yield of DSB clusters induced by heavier ions as C, N, O starts decreasing as a function of LET before the maximal LET is reached (Fig. 5). In both cases hooks in the curves connecting simulation points of damage vs. LET appear at the distal end of the Bragg peak. Analytical functions describing damage evolution as a function of LET can be introduced, at the price of ignoring the ambiguity of two different starting energies giving rise to energy depositions with the same LET. For DSB cluster induction, however, this ambiguity is negligible, and LET is a good indicator of damage over the full range of initial energies.

Within the proposed approach, the calculation scheme of Fig. 6 is finally adopted for the evaluation of neutron induced DNA damage using damage induction by secondary species (at LET values dependent on neutron energy), further weighted by their corresponding contribution to the total neutron dose. Evolution of neutron induced DNA damage endpoints with neutron energy necessarily depends on the evolution of the same endpoint as a function of LET for secondary charged particles (Fig. 7). In particular, the DSB cluster yield for charged particles shows a rise and fall as a function of LET similar to what is observed in typical RBE vs. LET curves for cell survival and related endpoints, showing overkilling effects. The yield of DSB clusters therefore correlates with cell survival for irradiation with charged particles, and, as a consequence, this remains true when neutron induced DSB clusters are obtained as a result of the coupling.

In order to compare the results of the simple phenomenological approach using y* as an indicator of effectiveness and of the full *ab-initio* approach for the evaluation of neutron induced DNA damage we finally give two possible RBE models.

A tentative neutron RBE is simply obtained dividing neutron y* by the corresponding value for a photon exposure of the phantom, always simulated with PHITS. This RBE model is by construction phenomenological, since it needs as an input from the systematics the saturation parameter y_0_. Resulting RBE values depend on the choice of this saturation parameter and on the *a priori* knowledge of the dependence of survival related RBE on linear energy transfer. Possible values for three different choices of y_0_ = 100, 150 and 200 keV/μm are collected in Fig. 8a. As the saturation parameter is increased, RBE values become higher and the second peak becomes more evident.

By the adoption of the approach proposed in this work instead, the neutron RBE model for DNA damage induction (damage yield by neutrons divided by damage yield by photons, obtained with PARTRAC) fully grounds on the results of this mechanistic study, starting from physical interactions. RBE values for DSB cluster induction are given in Fig. 8b. To our knowledge, this is the first neutron RBE model for DNA damage induction with *ab-initio* calculations starting from physical interactions.

As it can be seen comparing the results of the two models, substantial agreement is found between the purely mechanistic and the phenomenological approach, when an appropriate choice for y_0_ is made. The RBE model based on clustered DNA damage induction therefore offers a justification of the phenomenological approach, besides offering information on neutron induced DNA damage. Such information can be useful *per se*, as input for further modeling and experimental benchmark of modeling results with measurements of radiobiological endpoints. Predictions of neutron RBE obtained with the two models presented in this work can indeed be tested against results of radiobiological measurements, once a software replica of the experimental setups is obtained, including the relevant information on the secondary charged particle fields induced in the exposed biological samples^39^.

In both panels of Fig. 8 we also report for a qualitative comparison current standards for radiation weighting factors. Even if a quantitative comparison is not our aim, weighting factors are agreed upon by regulatory commissions on the basis (among other information, when available) of experimental studies on RBE. They provide therefore a useful term of comparison for RBE models proposed in this work. From the comparison we can conclude that simulated RBE values show rises and falls as a function of neutron energy in a coherent way with radiobiological datasets used for w_R_ evaluation. Absolute values are found to be close to ICRP and US.NRC standards, and generally intermediate between the two standards in the region of maximal effectiveness around 1 MeV. The importance of the second peak, for neutron energies around 20 MeV is also put in evidence: cells exposed to neutrons of similar energies would need to survive a highly complex DNA damage, which implies a relatively higher risk of misrepair and future transformation per surviving cell. The successful qualitative comparison of our RBE model predictions to neutron w_R_’s confirms the well-known important role for clustered DNA damage in driving the biological effectiveness^15^,^16^,^17^, with the understanding that the overall response to the radiation insult depends on a variety of factors at the cellular/tissue/organ level, and that is hardly conceivable to model it with a purely mechanistic approach. Dedicated radiobiological measurements can be foreseen to highlight *e*.*g*. differences in how different tissues cope with the same initial damage following neutron exposures as predicted by our RBE models^39^.

To conclude, the *ab-initio* approach presented in this work accomplishes the ambition of tracing back the origin of neutron biological effectiveness as a function of energy to physical interactions, choosing DNA damage as a representative endpoint of cellular effects. This work brings together the potential of transport calculations of neutrons through matter and of biophysical models predicting cellular damage with mechanistic approaches. Studies of this kind on early and late responses to different radiation types (but also doses, dose rates, *etc*.) starting from physical interactions are needed in the field^40^, as they deliver a fundamental insight into mechanisms of cellular radiation response and allow for a correct interpretation of the results of radiobiological measurements and for their applications in radiation protection and radiation therapy.

In particular, understanding the mechanisms at the basis of damage induction by neutrons generated during particle therapy (and high-energy photon therapy) should help scientists reassess the safety of possible radiotherapy alternatives^8^,^9^. An example of a roadmap to be followed in this sense entails the need for predictions of neutron RBE at any location in patients treated with proton therapy: this is demonstrated to be feasible and the results presented in this work can serve as input for this purpose^41^. In perspective, when such information is coupled to predictions of out-of-field neutron doses in the treatment^42^ and tissue-specific factors, a local quantification of the risk of second primary cancer occurrence can be achieved. A risk model of this kind has the potential to be tested and further improved thanks to prospective and retrospective epidemiological data from the follow-up of patients treated with proton therapy.

## Additional Information

**How to cite this article**: Baiocco, G. *et al*. The origin of neutron biological effectiveness as a function of energy. *Sci. Rep.* **6**, 34033; doi: 10.1038/srep34033 (2016).

## Supplementary Material

Supplementary Information

## References

[b1] Hall E. J. & Giaccia A. J. In Radiobiology for the Radiologist 7th edn (eds Lippincot W. &. W. ) Ch. 1 (Wolters Kluwer, 2011).

[b2] Caswell R. S. & Coyne J. J. Interaction of Neutrons and Secondary Charged Particles with Tissue: Secondary Particle Spectra. *Radiat*. *Res*. 52, 3 448–470 (1972). 4646440

[b3] ICRP. The 2007 Recommendations of the International Commission on Radiological Protection. ICRP Publication 103. Ann. ICRP 37 **(2–4)** (2007). 10.1016/j.icrp.2007.10.003 18082557

[b4] Health Risks from Exposure to Low Levels of Ionizing Radiation: BEIR VII Phase 2. National Academies Press (2006). 25077203

[b5] ICRP. 1990 Recommendations of the International Commission on Radiological Protection. ICRP Publication 60. Ann. ICRP 21 **(1–3)** (1991). 2053748

[b6] ICRP. 2003. Relative Biological Effectiveness (RBE), Quality Factor (Q), and Radiation Weighting Factor (w_R_). ICRP Publication 92. Ann. ICRP 33 **(4)** (2003). 10.1016/s0146-6453(03)00024-1 14614921

[b7] NRC Regulations Title 10, Code of Federal Regulations: 10 CFR. § 20.1004 (2014).

[b8] Ottolenghi A., Smyth V. & Trott K. R. Assessment of cancer risk from neutron exposure—The ANDANTE project. *Radiat*. *Meas*. 57, 68–73 (2013).

[b9] Ottolenghi A., Baiocco G., Smyth V. & Trott K. R. The ANDANTE project: a multidisciplinary approach to neutron RBE. *Rad*. *Prot*. *Dosim* . 166, 311–315 (2015). 10.1093/rpd/ncv158 25870432

[b10] Seth I., Schwartz J. L., Stewart R. D., Emery. R., Joiner M. C. & Tucker J. D. Neutron Exposures in Human Cells: Bystander Effect and Relative Biological Effectiveness. Plos One 9, 6, e98947 (2014). 24896095 10.1371/journal.pone.0098947 PMC4045982

[b11] Gajendiran N., Tanaka K. & Kamada N. Comet assay to assess the non-target effect of neutron-radiation in human peripheral blood. *J*. *Radiat*. *Res*. 42(2), 157–163 (2001). 11599882 10.1269/jrr.42.157

[b12] Sato T. . Particle and heavy ion transport code system, PHITS, version 2.52. *J*. *Nucl*. *Sci*. *Technol*. 50(9), 913–923 (2013).

[b13] Alloni D., Campa A., Friedland W., Mariotti L. & Ottolenghi A. Track structure, radiation quality and initial radiobiological events: considerations based on the PARTRAC code experience. *Int*. *J*. *Radiat*. *Biol*. 88 **(1–2)**, 77–86 (2012). 21957961 10.3109/09553002.2011.627976

[b14] Friedland W., Dingfelder M., Kundrát P. & Jacob P. Track structures, DNA targets and radiation effects in the biophysical Monte Carlo simulation code PARTRAC. Mutation Research 711, 28–40 (2011). 21281649 10.1016/j.mrfmmm.2011.01.003

[b15] Goodhead D. T. Initial events in the cellular effects of ionizing radiations: clustered damage in DNA. Int J Radiat Biol 65(1), 7–17 (1994). 7905912 10.1080/09553009414550021

[b16] Ward J. F. The complexity of DNA damage: relevance to biological consequences. *Int*. *J*. *Radiat*. *Biol*. 66, 427–432 (1994). 7983426 10.1080/09553009414551401

[b17] Georgakilas A. G., O’Neill P. & Stewart R.-D. Induction and repair of clustered DNA lesions: what do we know so far? *Radiat*. *Res*. 180(1), 100–109 (2013). 23682596 10.1667/RR3041.1

[b18] Gerlach R., Roos H. & Kellerer A. M. Heavy Ion RBE and Microdosimetric Spectra. *Radiat*. *Prot*. *Dosimetry* 99 **(1–4)**, 413–418 (2002). 12194343 10.1093/oxfordjournals.rpd.a006821

[b19] Pihet P., Menzel H. G., Schmidt R., Beauduin M. & Wambersie A. Biological Weighting Function for RBE Specification of Neutron Therapy Beams. Intercomparison of 9 European Centres. *Radiat*. *Prot*. *Dosimetry* 31 **(1–4)**, 437 (1990).

[b20] Stewart R. D. . Rapid MCNP simulation of DNA double strand break (DSB) relative biological effectiveness (RBE) for photons, neutrons, and light ions. Physics in Medicine and Biology 60(21), 8249 (2015). 26449929 10.1088/0031-9155/60/21/8249

[b21] International Commission on Radiation Units and Measurements. Tissue Substitutes in Radiation Dosimetry and Measurement. ICRU Report 44 (1989).

[b22] Satoh D., Takahashi F., Endo A., Ohmachi Y. & Miyahara N. Calculation of dose contributions of electron and charged heavy particles inside phantoms irradiated by monoenergetic neutron. *J*. *Radiat*. *Res*. 49, 503–508 (2008). 18580044 10.1269/jrr.08009

[b23] Baiocco G., Alloni D., Babini G., Mariotti L. & Ottolenghi A. Reaction mechanism interplay in determining the biological effectiveness of neutrons as a function of energy. *Rad*. *Prot*. *Dosim* . 166, 316–319 (2015). 10.1093/rpd/ncv134 25848097

[b24] Sato T., Kase Y., Watanabe R., Niita K. & Sihver L. Biological dose estimation for charged-particle therapy using an improved PHITS code coupled with a microdosimetric kinetic model. *Radiat*. *Res*. 171, 107–117 (2009). 19138056 10.1667/RR1510.1

[b25] Horiguchi H., Sato T., Kumada H., Yamamoto T. & Sakae T. Estimation of relative biological effectiveness for boron neutron capture therapy using the PHITS code coupled with a microdosimetric kinetic model. *J*. *Radiat Res*. 56(2), 382–390 (2015). 25428243 10.1093/jrr/rru109 PMC4380055

[b26] International Commission on Radiation Units and Measurements. Microdosimetry. ICRU Report 36 (1983).

[b27] Kellerer A. M. In The Dosimetry of Ionizing Radiation (eds Kase K. R., Biärngard B. E., Attix F. H. ) Ch. 2 (Academic Press Inc, 1985).

[b28] International Commission of Radiation Units and Measurements (ICRU). Microdosimetry. Report No 36 (Bethesda, MD) (1983).

[b29] Leuthold G. & Burger G. Dose mean lineal energy for neutrons. *Rad*. *Prot*. *Dos* . 31 **(1–4)**, 223–226 (1990).

[b30] Lindborg L. & Nikjoo H. Microdosimetry and radiation quality determinations in radiation protection and radiation therapy. *Rad*. *Prot*. *Dos* . 143 **(2–4)**, 402–408 (2011). 10.1093/rpd/ncq390 21227959

[b31] Schmitt E., Friedland W., Kundrát P., Dingfelder M. & Ottolenghi A. Cross-section scaling for track structure simulations of low-energy ions in liquid water. *Rad*. *Prot*. *Dos* . 166, 15–18 (2015). 10.1093/rpd/ncv302 25969528

[b32] Friedland W., Jacob P., Paretzke H. G., Ottolenghi A., Ballarini F. & Liotta M. Simulation of light ion induced DNA damage patterns. *Radiat*. *Prot*. *Dosimetry* 122 **(1–4)**, 116–120 (2006). 17166872 10.1093/rpd/ncl451

[b33] International Commission on Radiation Units and Measurements. Stopping Power and Ranges for Protons and Alpha Particles. ICRU Report 49 (1993).

[b34] International Commission on Radiation Units and Measurements. Stopping of Ions Heavier Than Helium. ICRU Report (2005).

[b35] Kase Y. Microdosimetric measurements and estimation of human cell survival for heavy-ion beams. Radiat Res . 166(4), 629–638 (2006). 17007551 10.1667/RR0536.1

[b36] Norbury J. W. . Galactic cosmic ray simulation at the NASA Space Radiation Laboratory. Life Sciences in Space Research 8, 38–51 (2016). 26948012 10.1016/j.lssr.2016.02.001 PMC5771487

[b37] Heilbronn L. H. . Neutron yields and effective doses produced by Galactic Cosmic Ray interactions in shielded environments in space. Life Sciences in Space Research 7, 90–99 (2015). 26553642 10.1016/j.lssr.2015.10.005

[b38] Qtool: Calculation of Reaction Q-values and Thresholds, available at http://t2.lanl.gov/nis/data/qtool.html (Accessed: 1st August 2016).

[b39] ANDANTE Report Summary, available at http://cordis.europa.eu/result/rcn/182088_en.html (Accessed: 1st August 2016).

[b40] Belli M., Ottolenghi A. & Weiss W. The European strategy on low dose risk research and the role of radiation quality according to the recommendations of the “ad hoc” high level and expert group (HLEG). *Radiat*. *Environ*. *Biophys*. 49, 463–468 (2010). 20383517 10.1007/s00411-010-0284-2

[b41] Schneider U., Hälg R. A., Baiocco G. & Lomax T. Neutrons in proton pencil beam scanning: Parameterization of energy, quality factors and RBE. Phys. Med. and Biol. 61, 6231–6242 (2016). 27486057 10.1088/0031-9155/61/16/6231

[b42] Schneider U., Hälg R. A. & Lomax T. Neutrons in active proton therapy: Parameterization of dose and dose equivalent. Z. Med. Phys. in press: http://dx.doi.org/10.1016/j.zemedi.2016.07.001. 10.1016/j.zemedi.2016.07.001 27524678

