# Supplementary material for: The origin of neutron biological effectiveness as a function of energy
Source: Sci Rep. 2016 Sep 22;6:34033. doi: 10.1038/srep34033 (PMC5032018; doi:10.1038/srep34033)
Supplement: Supplementary Information [file srep34033-s1.pdf]

The origin of neutron biological effectiveness as a function of energy

G. Baiocco[1,*], S. Barbieri[1], G. Babini[1], J. Morini[1], D. Alloni[2,3], W. Friedland[4], P. Kundrát[4], E. Schmitt[4], M. Puchalska[5], L. Sihver[5], A. Ottolenghi[1]

[1] Department of Physics, University of Pavia, Pavia, Italy
[2] INFN, National Institute of Nuclear Physics, Sezione di Pavia, Pavia, Italy
[3] LENA, Laboratory of Applied Nuclear Energy, University of Pavia, Pavia, Italy
[4] Institute of Radiation Protection, Helmholtz Zentrum München – German Research Center for Environmental Health, Neuherberg, Germany
[5] Technische Universität Wien, Wien, Austria

[*] corresponding author: giorgio.baiocco@unipv.it

Supplementary Material

Evaluation of neutron RBE at different depths in the receptor. Neutron Relative Biological Effectiveness (RBE) is known to vary with the geometry of the biological target and, within a given large size receptor, with the precise location of the region of interest inside it. In this work, we simulated the exposure of an ICRU44 soft tissue spherical phantom (15 cm radius - ICRU sphere geometry) immersed in an isotropic field of monoenergetic neutrons with energies in the range 10^{-5} - 10^{3} MeV. We obtained results in terms of the neutron saturation corrected dose mean lineal energy y^* (with saturation parameter y_0=100, 150 and 200 keV/μm) and of neutron-induced clustered damage to the DNA (DSB clusters) in three different scoring regions, with spherical shape and with equal radii r=1.5 cm, but with centers positioned at different distances d with respect to the phantom center along a common diameter: the most internal scoring region (*inner*) is concentric to the phantom (d=0 cm), the mid-depth one (*intermediate*) is at d=7.5 cm and the most external (*outer*) is at d=13.5 cm, touching the surface of the phantom. We also evaluated y^* and DSB cluster induction for a reference 220kV photon field. Two possible RBE models are proposed: RBE(y^*) is obtained by the ratio of neutron to photon saturation corrected dose mean lineal energies; RBE (DSB cluster) by the ratio of neutron- to photon-induced DSB cluster yields. Neutron RBE values are given as a function of primary neutron energy, irrespective of how such energy is modified during the interactions in the phantom. In the paper, RBE results are only given in the most external scoring regions, where neutron energy in the region is closer to nominal initial neutron energy for an external irradiation. Supplementary results presented here in Fig. S1 show how predictions of neutron RBE with the two models are modified when deeper-seated scoring regions are considered as targets of interest. By plotting such RBE values always as a function primary neutron energy, and when comparing to results for the most external scoring region and with the current standards for neutron radiation protection factors as *e.g.* ICRP w_R's, we can put in evidence the fact that an unambiguous local RBE evaluation throughout an exposed target needs to be performed associated to a local evaluation of neutron energy at the point of interest.

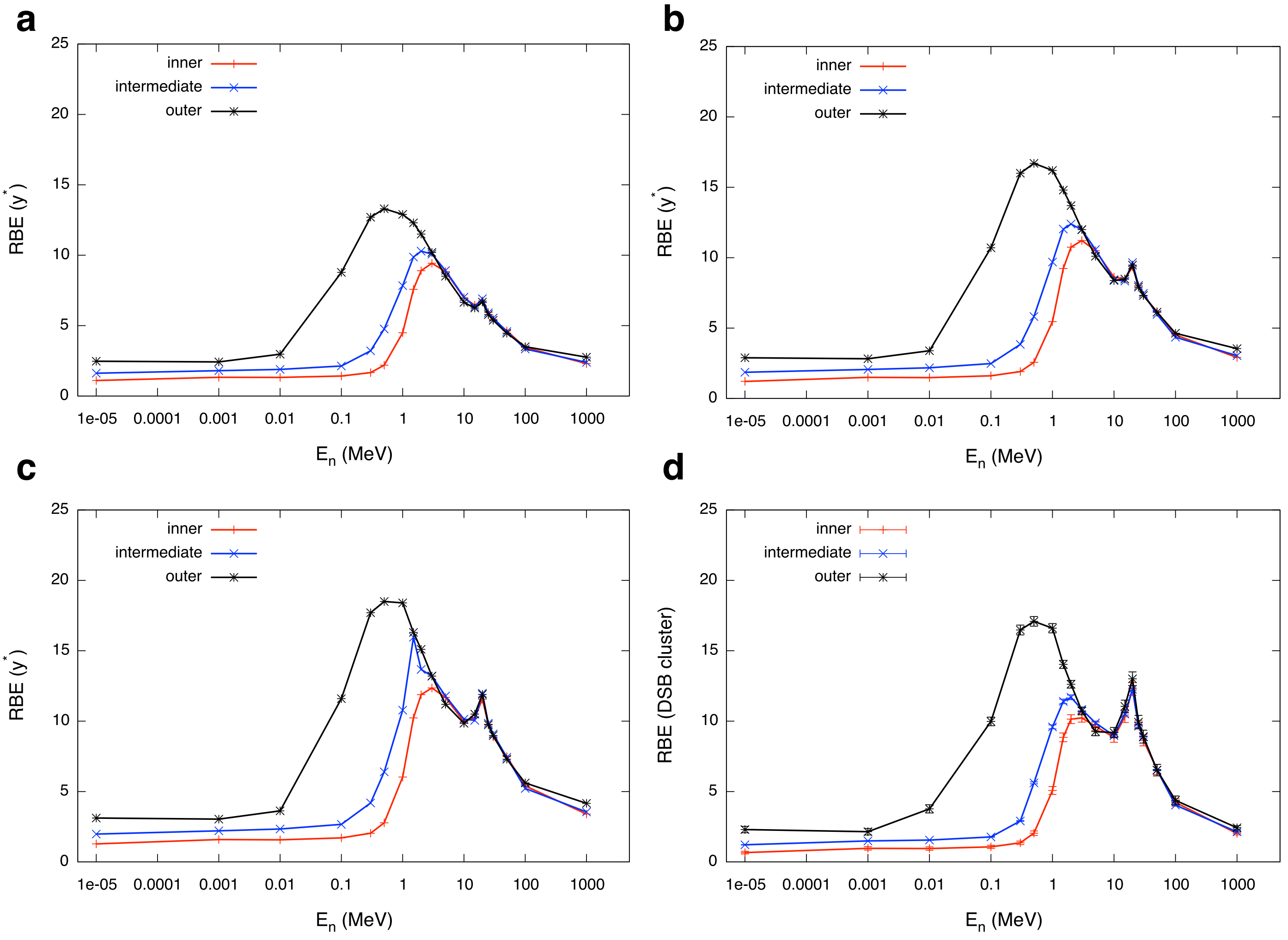

Figure S1. Model results for neutron RBE as a function of their energy and location in the phantom. Neutron RBE as a function of primary neutron energy in the three scoring regions (*inner*, *intermediate* and *outer*) and evaluated from: (a) to (c), ratio of saturation-corrected dose-mean lineal energies with y_0 = 100, 150 and 200 keV/μm, respectively; (d) DSB cluster induction. Lines are drawn to guide the eye. Error bars on RBE from saturation-corrected dose-mean lineal energies come from standard deviations among different PHITS runs for neutron and X-ray y^* values, and are within symbols. Error bars on RBE on from DSB cluster induction come from errors on the DSB cluster yields for neutrons and X-rays.
